# Comparison of suicide risk and other outcomes among boys and girls who self-harm

**DOI:** 10.1007/s00787-020-01490-y

**Published:** 2020-02-13

**Authors:** Anna Ohlis, Johan Bjureberg, Paul Lichtenstein, Brian M. D’Onofrio, Alan E. Fruzzetti, Martin Cederlöf, Clara Hellner

**Affiliations:** 1 Centre for Psychiatry Research, Department of Clinical Neuroscience, Karolinska Institutet, Stockholm, Sweden; 2 Stockholm Health Care Services, Stockholm County Council, Stockholm, Sweden; 3 Department of Medical Epidemiology and Biostatistics, Karolinska Institutet, Stockholm, Sweden; 4 Department of Psychological and Brain Sciences, Indiana University, Bloomington, IN USA; 5 Department of Psychiatry, Harvard Medical School, Boston, MA USA

**Keywords:** Self-injurious behaviour, Suicide, Sex characteristics, Follow-up studies, Mental health services

## Abstract

Little is known about sex differences in outcomes of self-harm, and there are inconclusive results concerning the association between sex, self-harm, and suicide attempts. The aim of this study was to explore sex differences in outcomes of self-harm in adolescence. In this cohort study, all individuals (0–17 years) enrolled at the child- and adolescent mental health services (CAMHS) in Stockholm between 2001 and 2015 (*N* = 110,072) were followed in national registers from their last contact with the CAMHS, until end of 2015. Exposure was self-harm as reason for contact, outcome measures were: alcohol-/substance use disorder, psychiatric hospitalization, non-violent or violent crime, and suicide. Differences in outcomes rates between exposed versus unexposed males, and exposed versus unexposed females, were examined using Cox regressions, expressed as hazard ratios (HR) with 95% confidence intervals (CI). Median follow-up time was 5.8 years (Q1: 2.3 years; Q3: 9.7 years). Self-harm was documented in 2.2% (*N* = 1241) males and 8.7% (4716) females. Exposed individuals had higher HR for all outcomes as compared with unexposed individuals of their own sex. Exposed females had more pronounced risk for drug use disorder (HR 11.2; 95% CI 9.9–12.7) compared with exposed males (HR 6.5, 95% 5.2–8.0). Both males and females who had engaged in self-harm had elevated risks for future suicide. Adjusting for socio-economic status and age at start of follow-up only marginally affected the associations. Females and males with self-harm had similarly elevated risk for suicide, and self-harm was also an important risk marker for other adverse outcomes within both sexes.

## Introduction

Self-harm refers to all self-directed harmful acts, regardless of suicidal intent (e.g., self-cutting and self-poisoning) [1, 2], and is a major public health concern among young people worldwide [2]. Self-harm is slightly more commonly reported in females, has a peak in prevalence rates at age 15–17 (onset age usually 12–14 years of age) and declines in early adulthood [1–3]. During adolescence, self-harm is associated with impulsivity [4], anxiety, depression, antisocial behaviour, high-risk alcohol use, cannabis use, and cigarette smoking [5]. Longitudinal studies show that self-harm is a risk factor for future substance misuse [6–12], violent behaviour [7, 12], and other adverse outcomes, such as unemployment [6, 7, 10]. Furthermore, self-harm is one of the strongest predictors of death by suicide [13–16].

While both males and females engage in self-harm, studies on sex differences in terms of long-term outcomes are sparse and available findings are inconclusive. In general, suicide attempts are more common among females, while suicides are more common among males [15]. Some studies show no sex differences in the association between self-harm and suicidal ideation or attempts [17, 18], while others find the association between self-harm and suicide attempts only among females [19]. There is an emerging awareness about self-harm being common among young males, with a reported lifetime prevalence of over 26% [3], but it remains unclear whether self-harm in males is associated with other risk profiles than females. Increasing suicide rates among young persons [20], along with insufficient knowledge concerning sex differences in outcomes associated with self-harm, one of the strongest predictors of suicide, stress the importance of studying whether associated risks differ for males and females.

Except from a large multi-center study from England that included over 5000 individuals presenting to hospitals due to self-harm [21], most longitudinal cohort studies on self-harm involving clinical populations are based on small samples [1]. The largest outcome studies on self-harm are based on data from national registries, typically using World Health Organization’s International Classification of Diseases’ (ICD) codes [22] for identifying the target populations, combined with linkage to various other registries for identifying outcomes. Beckman and colleagues included all Swedes aged 20 between 2001 and 2005 in a register-based cohort study, in which exposure was defined as hospitalization between ages 10–20 due to a diagnosis of intentional self-harm according to the ICD-10 (codes X 60-84 and Y 10-34) [6]. In this cohort of nearly 500,000 individuals, the association between self-harm and death by suicide was roughly the same for both sexes [6]. Another Swedish cohort study that included more than 1.8 million Swedes (age 15–32) found self-harm to be an independent risk factor for violent criminality, with the strongest risk for females [12].

However, a limitation with studies based on ICD-codes is that only the more severe cases are usually included. Both of the previously mentioned studies used a registered record of an ICD-code (*ICD*-*9: E950*-*9 and ICD*-*10: X60*-*84, or; ICD*-*9: E980*-*9 and ICD*-*10: Y10*-*34*) in the Swedish National Patient Register as a measure for self-harm exposure. Within the clinical practice of Child and Adolescent Mental Health Services (CAMHS) in Stockholm (a metropolitan area with almost two million inhabitants), self-harm is not consistently assigned X- or Y-diagnoses, and, therefore, will not be noticed in the National Patient Register as such. Therefore, albeit having large samples, Swedish register studies using the X- or Y-diagnosis as exposure for self-harm will most likely focus only on the subgroup of patients who self-harm severely enough to receive medical attention, but may not include the adolescents with less severe versions of self-harm or suicidal behaviours, or those who are in early phases of self-harm processes. In a previous study, our group addressed this gap by making use of a patient cohort identified through a regional clinical care register of patients who had contacted the CAMHS in the Stockholm County, where exposure to self-harm was documented by a clinician as a reason for contact with the mental health services [7]. However, our previous study lacked sufficient statistical power to explore whether males and females who seek care for self-harm differ in terms of outcomes.

The aim of this study was to examine subsequent adverse outcomes (alcohol/substance misuse, psychiatric inpatient care, suicide, and criminality) associated with care contact due to self-harm during adolescence, in a large enough sample to explore sex differences.

## Methods

### Registers

All Swedish residents are at birth, or immigration, assigned a personal identification number. This number is used by different governmental agencies for collecting data, including the health care system and various national registers. The data are organized and held by the governmental agency, Statistics Sweden. In this study we used outcome data from: (1) the National Patient Register that holds information on all psychiatric in- and outpatient specialist care (with complete coverage since 2006), including diagnostic codes according to the ICD-10 1997–2015; (2) the National Crime Register that holds data on all criminal convictions in Swedish lower courts; (3) the Register of Persons Suspected of Offenses with data on suspicion of a crime after completed investigation by police or other authority; (4) the Multi-Generation Register with information on family relations; (5) the Cause of Death Register which holds information on all deaths and the registered cause of death; and (6) the Longitudinal Integration Database for Health Insurance and Labour Market Studies (LISA) that contains information on education and employment.

Data on exposure were collected from a regional care register (the Pastill Register) that holds data on each patient at CAMHS Stockholm. CAMHS provide specialist-level psychiatric care to all inhabitants under age 18 in the Stockholm County. Apart from the public CAMHS, there are also some private caregivers who are commissioned by the County Council, but approximately 90% of the care budget goes to the public CAMHS. The Pastill Register was introduced in 1999, and since 2001, it holds information on contact reason, treatment provided, mental disorders according to the ICD-10, psychotropic medications, psychosocial problems, and global functioning on each patient at CAMHS. Data on contact reason and psychosocial problems are manually documented in Pastill by the clinician, while the remaining data are derived directly from the digital charts. The Pastill Register was linked to Swedish national registers using the personal identification number.

### The study cohort

The study cohort consisted of all individuals who were enrolled at CAMHS in Stockholm between 2001 and 2015 (*N* = 110,072). The cohort was followed for the outcomes from the last registered contact with CAMHS until the end of 2015.

### Exposure

Exposure was defined as having self-harm assigned as one (of potentially several) contact reason(s) in the Pastill Register. Contact reason was assigned by the responsible clinician at the time. During the early phases of the observation period, there was no specific documentation in the Pastill Register on suicidal intent; thus, individuals categorized as exposed could have presented with either non-suicidal or suicidal self-harm.

### Outcomes

The outcome measures in the national registers were (1) a diagnosis of alcohol or substance use disorder (ICD-10; F10-F19); (2) an inpatient psychiatric care (regardless of reason); (3) having been suspected or convicted of a non-violent or violent crime; and (4) death by suicide.

### Covariate

Analyses were adjusted for the potential effect of socioeconomic status, using the mother’s highest level of education as a proxy, and for age at start of follow-up (last registered contact with CAMHS).

### Statistical analyses

Differences in outcomes rates were analysed between: (1) exposed males versus unexposed males; and (2) exposed females versus unexposed females. Differences in outcome rates were examined using Cox regressions and expressed as hazard ratios (HR) with 95% confidence intervals (CI). The analyses were first made without adjustments (crude), then with adjustments for socioeconomic status and age at start of follow-up. The proportional hazards assumption was not violated in any analyses (tested with Stata’s estat phtest, which tests the proportional hazards assumption on the basis of Schoenfeld residuals).

### Supplementary analyses

Substance use disorder was further divided into separate drug classes (opiates, cannabis, sedatives/hypnotics, hallucinogens, stimulants, solvents and mixed substance use). Differences in outcomes rates after adjustments were analysed as above.

## Results

All results are presented in Table 1. Median follow-up time was 5.8 years (Q1: 2.3 years; Q3: 9.7 years). Self-harm as contact reason was documented in 2.2% (*N* = 1241) of all male and 8.7% (4716) of all female patients. Exposed males and females had elevated hazard ratios for all outcomes as compared with unexposed patients of their own sex. Females with self-harm compared to females without self-harm had an excess risk for drug use disorder (HR 11.2; 95% CI 9.9–12.7) relative to males with self-harm versus males without self-harm (HR 6.5, 95% CI 5.2–8.0). After adjusting for socioeconomic status and age at start of follow-up, all associations were somewhat diminished, but remained significant. Both males and females who had engaged in self-harm had elevated risks for future suicide.

**Table 1:** Outcomes for males with self-harm compared to males without self-harm, and females with self-harm compared to females without self-harm

	Males				Females			
	SH	Unexposed	HR (95% CI)		SH	Unexposed	HR (95% CI)	
	*N* (%)	*N* (%)	Crude	Adjusted^a^	*N* (%)	*N* (%)	Crude	Adjusted^a^
Individuals	1241 (2.2)	54,889 (97.8)	–	–	4716 (8.7)	49,226 (91.3)	–	–
Alcohol	112 (9.0)	2174 (4.0)	8.0*** (6.6–9.8)	6.1*** (4.9–7.5)	582 (12.3)	2663 (5.4)	8.6*** (7.8–9.5)	7.5*** (6.8–8.3)
Drugs	97 (7.8)	1964 (3.6)	6.5*** (5.2–8.0)	5.0*** (4.1–6.3)	400 (8.5)	1493 (3.0)	11.2*** (9.9–12.7)	9.7*** (8.5–11.0)
Hosp.	210 (16.9)	2771 (5.1)	8.6*** (7.4–9.9)	7.2*** (6.2–8.4)	1031 (21.9)	3910 (7.9)	9.5*** (8.8–10.2)	8.5*** (7.9–9.2)
Suicide	3 (0.2)	56 (0.1)	10.7*** (3.2–35.2)	4.7* (1.1–20.1)	10 (0.2)	58 (0.1)	9.1*** (4.3–19.1)	7.8*** (3.7–16.7)
Non-violent crime	216 (17.4)	7141 (13.0)	4.8*** (4.2–5.6)	3.6*** (3.1–4.2)	568 (12.0)	4379 (8.9)	5.9*** (5.3–6.4)	5.1*** (4.6–5.6)
Violent crime	95 (7.7)	3087 (5.6)	4.3*** (3.5–5.3)	3.2*** (2.6–4.0)	195 (4.1)	1093 (2.2)	6.1*** (5.2–7.2)	5.4*** (4.5–6.4)

Risk estimates are presented as hazards ratios (HR) with 95% confidence intervals (CI)

*Abbreviations*: *SH* self-harm, *Hosp*, hospitalization

^a^Adjusted for socioeconomic status and age at start of follow-up

*p ≤ .05; **p ≤ .01; ***p ≤ .001

### Supplementary analyses

All results are presented in the appendix, Table 2. HR for hallucinogens use among males could not be calculated due to low prevalence.

## Discussion

In this study, we investigated adverse outcomes in a clinical cohort of males and females who contacted care within the specialized mental health services in Stockholm, where a clinician had documented self-harm as one of the contact reasons. The main finding was that males as well as females exposed to self-harm before age 18 had similar—and higher—risk profiles with regard to adverse outcomes compared to patients without documented self-harm. Both males and females with self-harm had elevated risks for alcohol and drug misuse, subsequent psychiatric inpatient care, criminality (non-violent as well as violent), and suicide, as compared with other patients.

Our results provide further evidence of the associations between self-harm and later adverse outcomes previously found in a study with a smaller sample of former CAMHS patients [7], as well as in two cohort studies with data from the Swedish National Patient Register; one cohort (age 15–32) using X- or Y-diagnosis according to ICD-10 to define exposure [12], and the other using self-harm leading to hospitalization (X- or Y-diagnosis) to define exposure [6]. Mars and colleagues found similar associations in a population-based cohort study of British youth [10]. Thus, several studies confirm the association between self-harm and later adverse outcomes. Our study also indicates that in a younger cohort, with likely less severe self-harm (including not only those who had been assigned an X- or Y-diagnosis), the effect sizes were quite large. From a clinical standpoint, and given that these serious outcomes (i.e., suicide, criminality, substance- and alcohol misuse, hospitalization) occur already in early adulthood, these findings emphasize that young individuals presenting to care with identified self-harm need to be offered appropriate interventions at an early stage. The elevated risks for adverse outcomes among self-harming patients of both sexes cannot be ignored and more efficient prevention strategies, assessment procedures, as well as treatments are necessary. Adolescence is a crucial moment in time for interventions, not least, because the adolescent is still under supervision from parents, school, or other societal support, which can act on behalf of the adolescent, for example as a help-seeker or supporter.

The other important aspect of this study is that there was a risk of later adverse outcomes associated with self-harm within each sex, regardless of general differences and similarities between sexes. Although more males than females commit crimes, self-harming male patients had over four times higher risks of being convicted of a crime than male patients without self-harm, and for self-harming females that risk was nearly six times higher. Previous research show that females with SH exhibit high risks for violence [12] and substance misuse [9]. A possible reason could be that SH in females is an indication of borderline personality disorder (BPD), a condition associated with aggression and drug use. Yet, in a previous longitudinal study on Swedish data, the association between SH and violent offense [12] was to a greater extent explained by substance use disorder rather than by BPD only. This of course could be due to BPD not being properly diagnosed, but it is also possible that SH, drug misuse and violence are associated independently of BPD. From a clinical perspective, risk behaviours across the entire spectrum need to be assessed regardless of sex and incorporated in the treatment plans. Another clinically important finding was that the association between self-harm and later suicide was present in both sexes.

Previous research shows that across studies, women are more likely to report a history of self-harm than men, with larger sex differences for clinical samples [3]. This raises the question of whether self-harming males are less prone to seeking help from the health care system and/or if they are adequately assessed for self-harm. It has also been suggested that males may present with a different clinical picture, including lower reported severity levels of self-harm correlates [23]. Self-harm has a history of being thought of as a problem mainly affecting females [24], but reporting levels may also be influenced by perceived stigma [25, 26] as well as professional bias based on assumptions of sex difference. It is possible that SH in males to a larger extent run undetected or is misconceived, diminishing the strength of the association between SH in males and the different outcomes. Regardless, our data clearly point out that SH is a risk factor for adverse outcomes in both sexes, which could imply that SH may serve as a marker of problems with impulsivity and emotion dysregulation, both associated with, e.g., borderline personality disorder, substance use disorder and aggression. Importantly, given the elevated risks across sexes, SH screening, assessment, and treatment planning procedures need to be sex neutral.

This study may add to the knowledge concerning self-harm and sex; however, the study has limitations. Self-harming acts are often underestimated in clinical care [27] and despite using a more inclusive exposure measure than ICD-10 diagnoses, many self-harming acts were likely unrecorded in the present study, and it cannot be ruled out that there is a sex bias in recorded self-harm. In addition, there are no estimates of reliability for the clinician ratings. Furthermore, the distinction between male or female is done by biological sex at birth, and the study lacks data on gender identity. Hence, no conclusions can be made regarding differences in outcomes related to gender identity. Finally, the sample consisted of young people who contacted health care and the results may not generalize to non-help seeking populations.

## Appendix

See Table 2.

**Table 2:** Prevalence and risk estimates of substance use disorder by drug class, for males with versus without self-harm, and females with versus without self-harm

	Males with SH versus Males without SH			Females with SH versus Females without SH		
	SH	Unexposed	HR (95% CI)	SH	Unexposed	HR (95% CI)
	*N* (%)	*N* (%)	Adjusted^a^	*N* (%)	*N* (%)	Adjusted^a^
Individuals	1241 (2.2)	54,889 (97.8)	–	4716 (8.7)	49,226 (91.3)	–
Opiods	3 (0.2)	71 (0.1)	5.8 (1.8–19.3)**	16 (0.3)	100 (0.2)	7.5 (4.1–13.9)***
Cannabis	51 (4.1)	1285 (2.3)	3.5 (2.6–4.7)***	152 (3.2)	555 (1.1)	7.4 (6.0–9.1)***
Sedatives/hypnotics	11 (0.9)	173 (0.3)	7.2 (3.6–14.5)***	92 (2.0)	332 (0.7)	12.9 (9.8–17.1)***
Hallucinogens	1 (0.1)	46 (0.1)	–	8 (0.2)	21 (0.04)	26.3 (8.9–77.7)***
CNS stimulantia	13 (1.0)	187 (0.3)	10.2 (5.5–18.9)***	41 (0.9)	232 (0.5)	9.2 (6.3–13.5)***
Solvents	5 (0.4)	41 (0.1)	13.4 (5.0–35.5)***	14 (0.3)	39 (0.1)	9.9 (5.0–19.4)***
Mixed substance	15 (1.2)	325 (0.6)	7.7 (4.5–13.1)***	96 (2.0)	364 (0.7)	12.6 (9.6–16.6)***

Risk estimates are hazards ratios (HR) with 95% confidence intervals (CI)

*Abbreviations*: *SH* self-harm

^a^Adjusted for socioeconomic status and age at start of follow-up

**p* ≤ .05; ***p* ≤ .01; ****p* ≤ .001
